# Arginine Vasotocin Directly Regulates Spermatogenesis in Adult Zebrafish (*Danio rerio*) Testes

**DOI:** 10.3390/ijms25126564

**Published:** 2024-06-14

**Authors:** Maya Zanardini, Weimin Zhang, Hamid R. Habibi

**Affiliations:** 1 Department of Biological Sciences, University of Calgary, Calgary, AB 2500, Canada; maya.zanardini@ucalgary.ca; 2 School of Life Sciences, Sun Yat-sen University, Guangzhou 510275, China; lsszwm@mail.sysu.edu.cn

**Keywords:** vasotocin, AVP, spermatogenesis, zebrafish, ex vivo culture, male reproduction

## Abstract

The neuropeptide vasopressin is known for its regulation of osmotic balance in mammals. Arginine vasotocin (AVT) is a non-mammalian homolog of this neuropeptide that is present in fish. Limited information suggested that vasopressin and its homologs may also influence reproductive function. In the present study, we investigated the direct effect of AVT on spermatogenesis, using zebrafish as a model organism. Results demonstrate that AVT and its receptors (*avpr1aa*, *avpr2aa*, *avpr1ab*, *avpr2ab,* and *avpr2l*) are expressed in the zebrafish brain and testes. The direct action of AVT on spermatogenesis was investigated using an ex vivo culture of mature zebrafish testes for 7 days. Using histological, morphometric, and biochemical approaches, we observed direct actions of AVT on zebrafish testicular function. AVT treatment directly increased the number of spermatozoa in an androgen-dependent manner, while reducing mitotic cells and the proliferation activity of type B spermatogonia. The observed stimulatory action of AVT on spermiogenesis was blocked by flutamide, an androgen receptor antagonist. The present results support the novel hypothesis that AVT stimulates short-term androgen-dependent spermiogenesis. However, its prolonged presence may lead to diminished spermatogenesis by reducing the proliferation of spermatogonia B, resulting in a diminished turnover of spermatogonia, spermatids, and spermatozoa. The overall findings offer an insight into the physiological significance of vasopressin and its homologs in vertebrates as a contributing factor in the multifactorial regulation of male reproduction.

## 1. Introduction

Arginine vasotocin (AVT) is the non-mammalian counterpart of the mammalian arginine vasopressin (AVP) [1] (for clarity, the authors will follow the proposed ZFIN nomenclature available at https://zfin.org/, accessed on 1 October 2023). The AVP and its homologs are nonapeptides produced in different neuronal cell populations in the hypothalamus preoptic area (POA), and stored in the axon terminals of the neurohypophysis before their subsequent release into the circulation [2,3,4]. The AVP and its homologs regulate osmotic balance, stress, metabolism, and social behaviour [4,5,6]. However, increasing evidence from relatively few studies suggests that AVP and AVP receptors are expressed in ovaries and testes, indicating a possible reproductive role for these nonapeptides ([7]; for review see [8]). Five AVT receptors have been isolated in zebrafish, as follows: Avpr1aa and Avpr1ab (orthologous to human AVPR1A), Avpr2aa and Avpr2ab (orthologous to human AVPR2), and Avpr2l [9,10,11]. In particular, in male catfish (*Heteropneustes fossilis*), AVT and all the receptors are expressed in the testicular interstitial compartment [12]. Furthermore, AVT was shown to affect steroidogenesis in the ovaries of catfish [1]. Specifically, ovarian tissue incubated with increasing concentrations of AVT stimulated the production of estradiol (E2) and progestins in a dose-dependent and biphasic manner, depending on the stage of maturation [1,13]. Much less information is available on the role of AVP/AVT in males. Rodríguez and Specker (1991) first demonstrated that vasopressin and isotocin increased testosterone production in a dose-dependent manner in a rainbow trout (*Oncorhynchus mykiss*) testis in vitro [14]. The maximal response was observed at a concentration of 10 nM, increasing testosterone production by sixfold and fourfold, respectively, compared to the control [14]. Ramallo and colleagues showed that in vitro administration of exogenous vasopressin stimulated pituitary luteinizing hormone (LH) and follicle-stimulating hormone (FSH) secretion, as well as increased androgen production in the male cichlid fish *Cichlasoma dimerus* [15]. In male catfish (*Clarias magur*), treatment with AVT enhanced milt release by stripping and increased the expression of specific enzymes involved in the synthesis of steroids, namely star, 3bhsd, 17bhsd, cyp17a1a, and cyp11a1a [16]. However, mouse spermatozoa treated in vitro with high doses of AVP (10^−5^ and 10^−8^ M) displayed inhibition of sperm functions (sperm motility, capacitation, acrosome reaction) through the interaction of AVP with the receptor AVPR2 [17]. Overall, the role of AVP at the testicular level remains largely unclear.

Spermatogenesis is a highly coordinated process regulated by the hypothalamus-pituitary-gonad (HPG) axis [18,19,20]. In addition to the classic endocrine regulation, evidence demonstrates the significance of local autocrine/paracrine factors in regulating spermatogenesis and testicular function in different species [21,22,23,24,25,26,27,28]. In zebrafish, spermatogenesis occurs within spermatocysts formed when Sertoli cells surround one single spermatogonial stem cell (SSC) that proliferates and differentiates into many haploid spermatozoa [29,30]. The activity of SSC is critical to support spermatogenesis, as these cells can both self-renew to maintain a pool of stem cells or commit to spermatogenesis, becoming haploid germ cells. This balance between self-renewal and differentiation is crucial in maintaining spermatogenesis [31]. The decision for SSCs to differentiate is mediated by cell–cell communication. It marks the beginning of the mitotic phase, where several rounds of mitotic division lead to spermatogonia generations, identified as spermatogonia type A undifferentiated (Aund), type A differentiated (Adiff), and type B. In this phase, Fsh-induced growth factors from Sertoli cells are involved in the regulation of stem cell renewal, spermatogonia proliferation, and differentiation [31]. Studies have shown that both the gonadotropin-releasing hormone (GnRH) and gonadotropin-inhibitory hormone (GnIH) produced in testicular germ and somatic cells are components of a multifactorial mechanism that regulate gonadotropin-induced androgen production, spermatogonia proliferation, and spermatogenesis [24,25]. During the meiotic phase, type B spermatogonia differentiate into spermatocytes that undergo meiotic division, resulting in haploid spermatids. Spermatids mature into flagellated spermatozoa in the spermiogenic phase [30]. While levels of androgens are elevated throughout germ cell development, LH affects the spermiogenic phase by stimulating the germ cells and steroidogenesis [30]. Fallah and colleagues further demonstrated that the effect of LH was potentiated in the presence of GnRH isoforms (GnRH2 and GnRH3) in an androgen-dependent manner [24].

The objective of the present study was to investigate the direct action of AVT on basal spermatogenesis using an ex vivo culture of zebrafish testes. We focused on spermatogonia germ cell proliferation activity and cell development, androgen production by Leydig cells, and measurement of testicular transcript abundance, including germ cell markers, steroidogenic enzymes, and gonadotropin receptors. In addition, using RT-qPCR, we measured the transcript levels of *avt* and all five Avp receptor subtypes (*avpr1aa*, *avpr1ab*, *avpr2aa*, *avpr2ab*, *avpr2l*) in the brain and testis of zebrafish. The findings support the novel hypothesis that AVT stimulates short-term androgen-dependent spermiogenesis. However, its prolonged presence may lead to diminished spermatogenesis by reducing the proliferation of spermatogonia B, resulting in a diminished turnover of spermatogonia, spermatids, and spermatozoa.

## 2. Results

### 2.1. Transcript Levels for AVT and AVP Receptors

**Figure 1:**
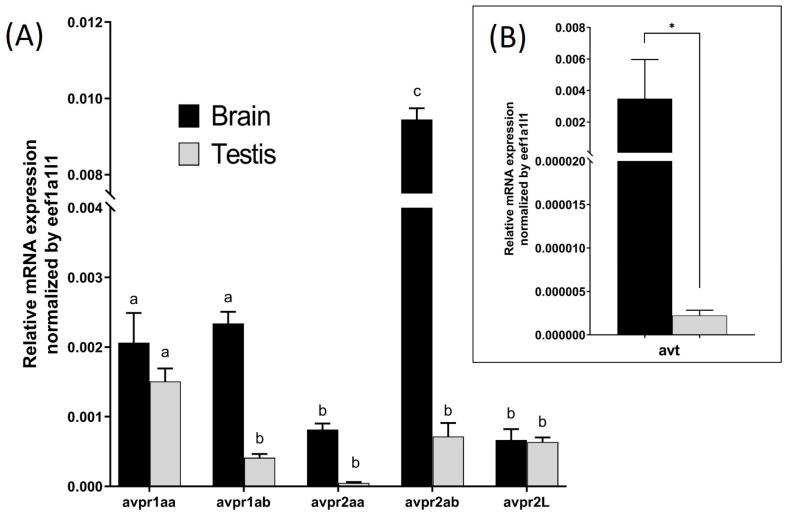
Relative transcript abundance of *avt* and Avp receptor subtypes in the testes and brain of zebrafish. Results show mRNA abundance of Avp receptor subtypes (*avpr1aa*, *avpr1ab*, *avpr2aa*, *avpr2ab*, *avpr2l*) (**A**) and vasotocin (*avt*) (**B**) in the adult zebrafish testis compared to the male brain. Columns represent basal expression levels from non-treated tissues (*n* = 5). Values were normalized with respect to *eef1a1l1*. Values with dissimilar superscripts are significantly different using a two-way ANOVA. Asterisk represents significant differences in transcript abundance of *avt* in the brain vs. testis, analyzed by the Mann–Whitney test.

Transcript abundance of *avt* (Figure 1B) and all five avp receptor subtypes (*avpr1aa*, *avpr1ab*, *avpr2aa*, *avpr2ab*, *avpr2l*) (Figure 1A) in the brain and testis of zebrafish were measured using *eef1a1l1* as an internal control. The relative abundance of the receptor subtypes within each tissue was generally higher in the brain compared to the testis (Figure 1A,B). In the brain, avpr2ab was found to be significantly more abundant than all other subtypes. This was followed by *avpr1aa* and *avpr1ab*, both of which were significantly more abundant than *avpr2aa* and *avpr2l* (Figure 1A). In the testis, *avpr1aa* was the most prevalent subtype, with its levels significantly exceeding those of the other subtypes.

### 2.2. Effect of AVT on Basal Spermatogenesis

**Figure 2:**
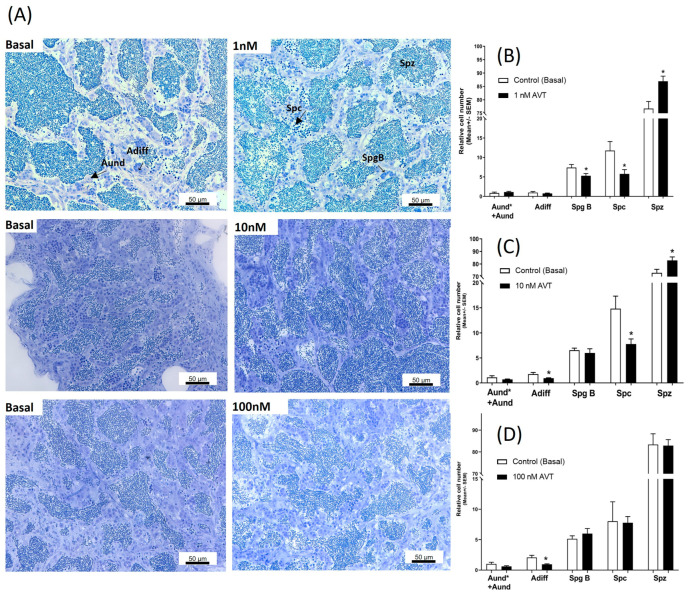
Dose-related effects of AVT on spermatogenesis. (**A**) Isolated contralateral testes were incubated separately, one as a control, the other with increasing concentrations of AVT. The arrangement of the figure reflects the way this experiment was conducted. The control for each concentration was from the same fish and different for different concentrations of AVT. Histological sections of zebrafish testes incubated ex vivo with or without (Basal) AVT for 7 days (**A**). Quantified cell types from testes incubated with 1 nM (**B**), 10 nM (**C**), and 100 nM (**D**) of AVT. The quantified results demonstrate the relative numbers of spermatogonia type A undifferentiated (Aund* + Aund), type A differentiated (Adiff), type B (SpgB), Spermatocytes (Spc), and Spermatozoa (Spz) for each concentration. Each treatment was analyzed with respect to its own (individual) control (contralateral testis). Asterisks represent significant differences between treatment and control, analyzed by Student’s *t*-test (*n* = 8–10), *p* < 0.05.

Morphological and stereological analysis provided information on the number of different cell types at the early and late stages of germ cell development following treatments with increasing concentrations of AVT. The relative number of spermatogonia type A undifferentiated (Aund* + Aund) was not affected by AVT treatments. Treatment of testes with 10 and 100 nM AVT significantly decreased the number of spermatogonia A differentiated (Adiff) (Figure 2B,C). No other changes were observed following AVT treatment at 100 nM (Figure 2D). At 1 nM, AVT significantly decreased the number of diploid spermatogonia type B and spermatocytes, but increased the number of haploid spermatozoa (Figure 2B). At 10 nM, AVT also decreased the number of spermatocytes and increased the number of spermatozoa (Figure 2C).

**Figure 3:**
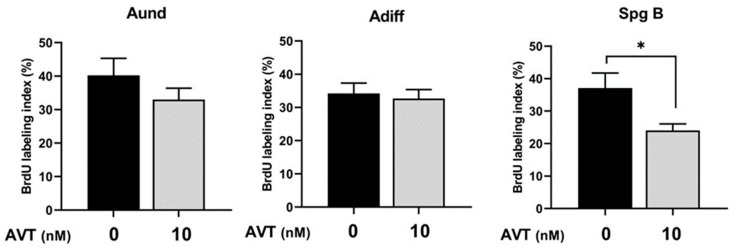
BrdU-labeling index of Aund, Adiff, and type B spermatogonia in basal conditions and in the presence of AVT (10 nM). BrdU was incorporated during the last 6 h of 7-day ex vivo testis culture. Bars show the mean ± SEM. Asterisks represent significant differences between treatment and control, analyzed by Student’s *t*-test (*n* = 5–7), *p* < 0.05.

To determine if changes observed in the diploid cells are due to altered mitosis, we measured the mitotic index using a BrdU incorporation assay. The BrdU assay was only performed for 10 nM AVT since, at this dose, we observed changes in Adiff, spermatocytes, and spermatozoa. Treatment with AVT (10 nM) did not change the mitotic index of type Aund and type Adiff spermatogonia, but significantly reduced the number of BrdU-positive type B spermatogonia (Figure 3), indicating a reduction in mitosis, leading to a reduction in the number of spermatocytes (Figure 2C).

#### 2.2.1. The Role of Androgens

**Figure 4:**
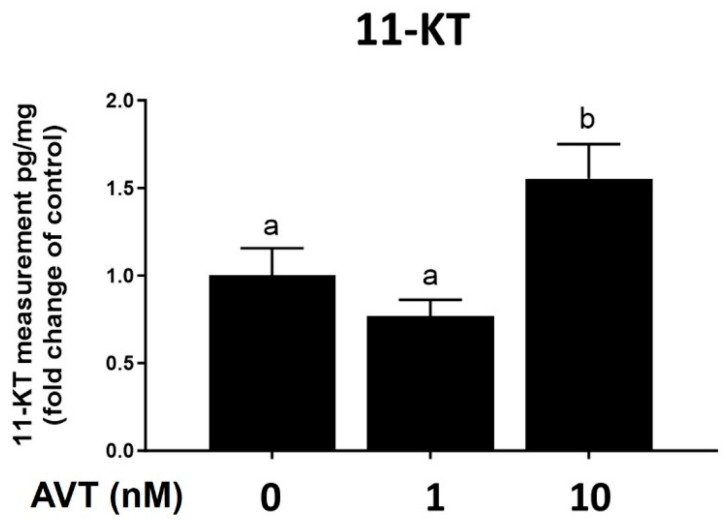
Quantification of 11-ketotestosterone in cultured testis medium after 7 days of treatment with 0, 1, and 10 nM vasotocin (AVT). Results are expressed as fold changes of their own (individual) control. Values are mean ± SEM of 6–8 replicate cultures. Different letters indicate significant differences between groups (one-way ANOVA followed by Tukey’s multiple comparison test, *p* < 0.05).

In the present study, we observed an increase in the AVT-induced number of spermatozoa at lower concentrations of 1 and 10 nM. Since the final stages of spermatogenesis are androgen-dependent [24,26,30], we tested the involvement of androgens in AVT-induced spermatozoa production by measuring the level of 11-KT and blocking the androgen receptors. The release of 11-ketotestosterone in the medium was measured following 7 days of treatment with 1 and 10 nM AVT. In this experiment, AVT treatment at 10 nM AVT significantly increased levels of 11-KT released by the testis (Figure 4).

**Figure 5:**
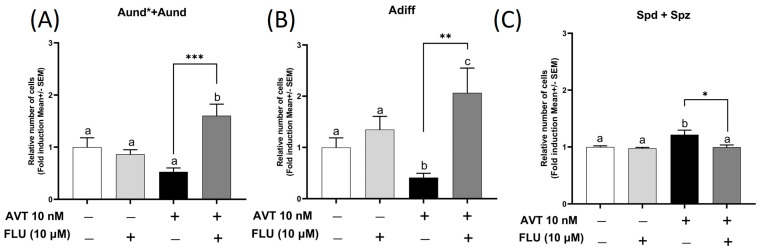
The effect of 10 μM of flutamide (FLU), alone or in combination with 10 nM vasotocin (AVT), on (**A**) spermatogonia type Aund* + Aund, (**B**) Adiff, and (**C**) haploid spermatids and spermatozoa, following 7 days of ex vivo culture. Cells were counted in the control vs. treatment groups, and each treatment group was normalized against its control and expressed as fold change (mean ± SEM; *n* = 8–10). Values displaying different symbols are significantly different (one-way ANOVA followed by Tukey’s multiple comparison test, * *p* < 0.05, ** *p* < 0.01, *** *p* < 0.001).

Concomitant treatment of 10 nM AVT with an androgen receptor antagonist flutamide (FLU) completely blocked the observed AVT-induced response. Treatment with FLU inhibited the AVT-induced increase in haploid cell populations, indicating that the effect of AVT on spermatogenesis involves androgen receptor activation (Figure 5C). Interestingly, the presence of flutamide also affected early stages of spermatogenesis, and its presence with AVT increased the number of spermatogonia type Aund* + Aund and type Adiff (Figure 5A,B), compared to the control and AVT alone.

#### 2.2.2. Effect of AVT on Transcript Abundance of Selected Genes Involved in Spermatogenesis

**Figure 6:**
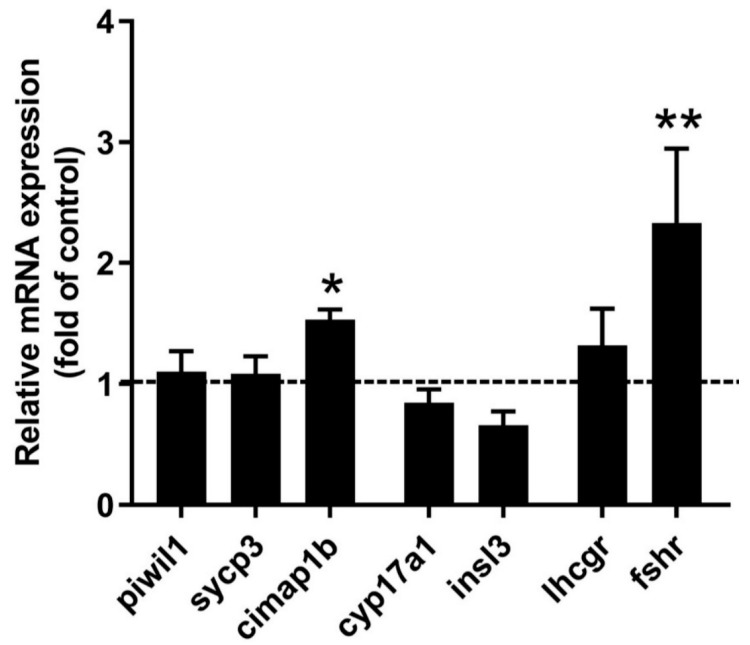
Relative transcript abundance of *piwil1*, *sycp3*, *cimap1b* (previously *odf3b*), *cyp17a1*, *insl3*, *lhcgr*, and *fshr* in testes sampled after 48 h of ex vivo treatment with 10 nM AVT. The dotted line represents the control group. Each treatment is presented as fold change to the control. The results were analyzed with respect to their own (individual) control using Student’s *t*-test (*n* = 8–10). Values displaying different symbols are significantly different compared to the control (* *p* < 0.05, ** *p* < 0.01).

We measured transcript abundances of specific germ cell markers, namely *piwil1* (marker for spermatogonia type Aund and Adiff), *sycp3* (marker for spermatocytes), and *cimap1b* (previously known as *odf3b* or *shippo1*; marker for spermatids and spermatozoa); the steroidogenic enzyme *cyp17a1*; the Leydig cells product *insl3* (involved in promoting differentiation and proliferation of type Aund spermatogonia); and gonadotropin receptors *lhcgr* and *fshr*. Treatment with 10 nM AVT significantly increased basal transcript levels of *cimap1b* and *fshr*, while having no effect on the other selected genes (Figure 6).

## 3. Discussion

While the role of vasopressin (AVP) as a regulator of osmoregulation, stress, and social behaviour has been well documented, only a few studies have focused on the effect of this nonapeptide on reproduction. In particular, very limited information is available on the role of AVT in the regulation of male reproduction in teleost [2,15,32,33,34,35,36,37]. The present study provides novel information about the role of AVT on zebrafish spermatogenesis. Using RT-qPCR, we demonstrated the expression of AVT and all five AVP receptors within the zebrafish testis. Using histological and morphological analysis, we demonstrated the direct action of AVT on germinal cell proliferation in the zebrafish testis. The effects of AVT were androgen-dependent, since it stimulated the production of 11-KT, and its actions were completely blocked by concomitant treatment with the androgen receptor antagonist, FLU. The presence of the AVT transcript in the testis (Figure 1B) strongly suggested an autocrine/paracrine activity of the hormone. There is no information on the circulating levels of AVT in zebrafish. In flounder, reported AVT concentration is in the range of 2–10 fmol/mL [6]. In mammals, the plasma level of vasopressin is higher, in the range of 1–5 pmol/mL [36]. While we cannot totally rule out the systemic action of AVT of hypothalamic origin on testicular spermatogenesis, it is unlikely that the circulating levels of AVP/AVT reach the levels needed to exert direct action on the testis in mammals or fish based on the observed dose-related effects of AVT on testicular function in the present study. The results demonstrate lower transcript levels for AVT in the testis compared to the brain; however, this does not necessarily translate to lower AVT content in the testis. In this context, a higher level of AVT may be possible in the testis since this peptide is produced locally, as shown in the present study. We also observed a different expression profile of AVP receptors in the brain compared to the testis. The *avpr2ab* receptor is the most highly expressed in the male brain, while *apr1aa* appears to be the most abundant AVP receptor subtype in the testes of adult zebrafish. Previous studies conducted on zebrafish demonstrated that AVT has a lower affinity for the avpr1aa receptor (EC50 727 ± 338 nM), and a higher affinity for the avpr1ab receptor (EC50 2.79 ± 1.4 nM) [6,38].

To investigate the effects of AVT on spermatogenesis and testicular development, we incubated testes with the following three concentrations of AVT: 1, 10, and 100 nM, using an ex vivo culture system. The concentration range was chosen based on previous published work in catfish [37]. We initially tested a high concentration of AVT at 100 nM, but omitted it from subsequent studies since we did not see any response. While we do not have direct evidence, the observed response to 100 nM may have been caused by desensitization or overlap of the specificity normally observed at very high hormone concentrations. The two lower doses tested (1 nM and 10 nM AVT), which are more likely to reflect physiological levels, increased the production of haploid spermatozoa cells while reducing the number of spermatocytes. To further investigate the observed action of AVT on haploid cells, we measured 11-KT released in the medium. Treatment with 10 nM AVT significantly increased 11-KT production, suggesting that the observed actions of AVT may be mediated, in part, by androgens. To test this postulate further, we used flutamide (FLU), an androgen receptor antagonist. Concomitant treatment with FLU totally blocked the AVT-induced response, indicating that the observed actions of AVT on spermatogenesis are androgen-dependent. This is consistent with observations in other studies, demonstrating a stimulatory dose-dependent effect of AVP on testosterone production in the immature testis of rainbow trout and the testis of adult cichlid fish in vitro [14,15]. The observed stimulatory action of AVT on spermiogenesis and 11-KT production is consistent with a study that tested the acute actions of AVT in zebrafish, in vitro. Acute injection with AVT stimulated male zebrafish reproductive success and male courtship behaviour [2]. Concomitant administration with an AVT antagonist abolished male reproductive success without affecting male courtship behaviour [2] and endocrine indices, possibly linked to a synergistic action of nonapeptides on male pheromone release. In the latter in vivo study, however, acute treatment with AVT did not alter circulating androgen levels in zebrafish [2]. In the present study, treatments with 1 and 10 nM AVT reduced the number of type B spermatogonia and Adiff, respectively. The AVT-induced reduction in B spermatogonia and Adiff numbers were blocked in the presence of the androgen receptor antagonist, FLU. In fact, the number of Aund and Adiff increased, while the number of spermatozoa diminished in the presence of FLU, indicating that AVT-induced androgen production blocks early spermatogenesis while stimulating androgen-dependent spermiogenesis, although we cannot rule out the involvement of other pathways [31].

Based on the present findings, we propose that chronic action of AVT will stimulate Leydig cells to produce 11-ketotestosterone, which stimulates spermatocytes to enter meiosis and differentiate into spermatozoa, resulting in an initial increase in spermatozoa production in the testis. However, the chronic treatment with AVT resulted in a reduction of Aund, Adiff, and type B spermatogonia, which could lead to a diminished number of early spermatogonia germs cells and spermatozoa. In the present study, short-term treatment with AVT increased the *cimap1b* transcript level, which is a specific marker for haploid cells, in accord with the observed increase in spermatogenesis. However, AVT did not influence levels of the other markers for Aund and Adiff *(piwil1*) or spermatocytes (*sycp3*). One possible reason for this could be the increased production of 11-KT induced by AVT, which could reduce or diminish the activity of early spermatogonial cells expressing *piwil1* and *sycp3.* This postulate is consistent with an observation in zebrafish treated with an insecticide beta-cypermethrin that increased androgen production by downregulating testicular expression of *cyp19a*, *nanos2*, *piwil1*, *dazl,* and *sycp3* [39].

Interestingly, the transcript level of the FSH receptor (*fshr*), but not the LH receptor (*lhcgr*), increased two-fold in the presence of AVT. In fish, FSH has a potent steroidogenic activity, and its receptor is expressed by both Leydig and Sertoli cells [40,41,42,43,44]. Upregulation of the FSH receptor level may facilitate a response to circulating levels of FSH. It is important to note that this study has some technical limitations, particularly when it comes to evaluating the long-term effects of hormones in the ex vivo culture system. The culture of the testis ex vivo cannot be extended over a 7-day period due to diminished viability beyond the culture period. It would be imperative to investigate the prolonged action of this nonapeptide on testicular development in vivo.

## 4. Material and Methods

### 4.1. Animals

Adult male zebrafish (TL strain), bred and raised in the Department of Biological Sciences aquatic facility at the University of Calgary (Calgary, AB, Canada), were used as model organisms. Fish were maintained in 10 L tanks in a recirculating system with controlled conditions (water temperature 28 °C, pH 7.6, and conductivity 750 μS) and a photoperiod of 14 h of light and 10 h of dark. Fish were fed twice daily with the adult commercial food Zeigler^®^ (Pentair Aquatic Habitats). Procedures were performed according to the University of Calgary Animal Care guidelines (protocol# AC19-0160).

### 4.2. Hormones and Chemicals

The (Arg^8^)-Vasotocin peptide (AVT; CYIQNCPRG (C1–C6 bridge); molecular weight: 1051.22 g/mol; peptide purity > 95%) was purchased from ChinaPeptides Co., Ltd. (Shanghai, China). The AVT peptide was reconstituted with 1× PBS and stored at −20 °C until use. For ex vivo tissue culture, AVT aliquots were diluted with L-15 culture medium to reach the final concentrations of 1, 10, and 100 nM.

Flutamide (FLU) is a synthetic, non-steroidal anti-androgen compound (NSAA) known to be a selective androgen receptor (AR) antagonist. In this study, flutamide was purchased from Sigma-Aldrich (MilliporeSigma Canada Ltd., Oakville, ON, Canada), reconstituted with 100% ethanol and diluted to a concentration of 10 μM, based on previous works on zebrafish [26].

### 4.3. Ex Vivo Testis Culture

The ex vivo testis culture system consisted of a 24-well plate, with each well containing a 2% agarose block with a nitrocellulose membrane (25 µm of thickness and 0.22 µm of porosity) on top, covered with filtered–sterilized Leibovitz’s L-15 Medium (Thermo Fisher Scientific, Mississauga, ON, Canada) [25,29]. Testes were dissected from zebrafish and cultured on top of the nitrocellulose membrane for 48 h (short-term) or 7 days (long-term). From one fish, one testis was used as a control and incubated only with L-15 medium, while the contralateral testis was incubated with L-15 medium supplemented with AVT alone, flutamide alone, or a combination of AVT and flutamide, as indicated. Culture plates were incubated at 28 °C for 48 h (short-term culture) to measure transcript abundance, and for 7 days (long-term culture) to evaluate the effect of AVT on spermatogenesis and to measure 11-ketotestosterone production. The 7-day treatment underwent a complete medium change on the 3rd day. After incubation, testes were collected for histological, biochemical, and morphometric analysis. Mitotic turnover was assessed using the BrdU proliferation assay [25]. Experiments were repeated 6–10 times as indicated.

### 4.4. RNA Extraction and RT-qPCR

After the short-term (48 h) treatment, testes were pooled (n = 2), and the total RNA was extracted using the Phenol-Chloroform extraction method and a TRIzol reagent (Invitrogen, Burlington, ON, Canada) [45]. Purity and quantity were assessed using the Nanodrop 2000 spectrophotometer (Thermo Scientific, Waltham, MA, USA), followed by DNase I (RNase-free; Thermo Fisher Scientific) digestion, and cDNA synthesis using a High Capacity Multiscribe cDNA kit (Invitrogen, Burlington, ON, Canada). RT-qPCR was performed using SsoFast Eva Green Supermix (BioRad, Mississauga, ON, Canada), as previously described [20]. Briefly, reagents (10 μL of Supermix, 1.6 μL forward + reverse primers, 7.4 μL ultrapure water, 1 μL cDNA per well) were mixed and aliquoted into a qPCR plate. Reactions were run in 20 µL volumes in duplicates with the following protocol: 95 °C for 2 min, 95 °C for 10 s, primer specific temperature for 30 s, 95 °C for 10 s, and 65 °C to 95 °C for 5 s, increasing 0.5 °C each time for melt curve. Steps 2 and 3 are repeated 39 times. Cq values were measured using a Bio-Rad CFX96 TouchTM Real-Time PCR Detection System (Bio-Rad, Hercules, CA, USA). The mRNA levels of the targets (Cq) were normalized against the reference gene *eef1a1l1* (elongation factor 1, alpha 1, like 1) and expressed as fold induction of relative values of the control group, according to the 2^−(∆∆CT)^ method [46]. New primers were designed based on the zebrafish sequences available on GenBank (NCBI, https://www.ncbi.nlm.nih.gov/genbank, accessed on 15 April 2022; refer to Table 1 for forward and reverse primers), or synthesized using sequences published previously. To prove the amplification of the correct sequence of interest, PCR products were Sanger-sequenced at the Centre for Health Genomics and Informatics at the University of Calgary.

### 4.5. Quantification of Androgen Released (ELISA)

We measured 11-ketotestosterone (11-KT) in the culture medium using an Enzyme-Linked Immunosorbent Assay (ELISA) kit (Cayman Chemicals, Ann Arbor, MI, USA). Testes were individually weighed prior to the culture, and after 7 days the medium was collected and used for 11-KT quantification. A SpectraMax i3 microplate reader (Molecular Devices, San Jose, CA, USA), set at 405–420 nm, was used to spectrophotometrically measure the reaction product. The concentration of 11-KT was determined per mg of tissue and expressed as relative fold change with respect to the controls.

### 4.6. Histomorphometric Analysis

The histological preparations were carried out as previously described [26,47]. After the 7-day incubation period, testes were collected and fixed overnight in 1.6% paraformaldehyde, 2.5% glutaraldehyde, and PBS 1× (pH: 7.2). Following ethanol dehydration, tissues were embedded in Technovit 7100 (Heraeus Kulzer, Wehrheim, Germany). Sections of 3 μm thickness were cut using a Ralph Knife on a Reichert-Jung 2040 Autocut Rotary Microtome. During the cutting process, we used a systematic uniform random sampling (SURS) method [48] to avoid bias, i.e., one section every three cuts was collected and used for image analysis. Sections were then stained with 0.1% Toluidine Blue to detect and quantify germ cell populations, and bright field images were taken at 100× magnification. For each section, a total area of 20,000 µm^2^ was used to quantify germ cells using Fiji software (version 1.54f) [49]. Five sections per replicate were used for analysis.

### 4.7. BrdU Incorporation Assay

BrdU (Bromodeoxyuridine/5-bromo-2′-deoxyuridine) is a thymidine analogue incorporated into replicating DNA (s-phase cells), which was used in the present study to evaluate the proliferation activity of spermatogonia germ cells. The BrdU pulse of 100 μg/mL was given to testicular explants in the last 6 h of the long-term incubation period (7 days). Processing and immunostaining of the testes was performed as described by Fallah and colleagues [24]. Briefly, after fixation with methacarn (60% methanol, 30% chloroform, 10% acetic acid) and dehydration, testis explants were embedded in Technovit 7100 (Heraeus Kulzer, Wehrheim, Germany). Sections of 3 μm thickness were cut and subjected to antigen retrieval and blocking. Immunostaining of BrdU-positive cells was performed using the mouse monoclonal anti-BrdU antibody (1:40) (Abcam cat# ab8955) and the mouse-specific HRP/DAB (ABC) Detection IHC Kit (Abcam cat# ab64259). Sections were counterstained with Gill hematoxylin or 0.1% Toluidine Blue. For each section, an area of 20,000 µm^2^ was used to quantify the germ cells. In this area, BrdU-positive vs. BrdU-negative type A undifferentiated, differentiated, and type B spermatogonia were counted, and the mitotic index was evaluated [50].

### 4.8. Statistical Analysis

Data from histology, transcript level, and immunohistochemistry with BrdU were analyzed using unpaired *t*-tests. For data that did not meet the condition for the parametric *t*-test, the non-parametric Mann–Whitney test was used. One-way ANOVA, followed by Tukey’s post hoc multiple comparisons test, was used for the flutamide experiment and ELISA. Two-way ANOVA, followed by Sidak’s multiple comparison test, was used to compare the vasopressin receptor transcript levels in the brain and testis. Differences were considered significant when *p* < 0.05. All statistical tests were performed using GraphPad Prism 8.0 software (Graphpad Software Inc., La Jolla, CA, USA).

## 5. Conclusions

In summary, our findings demonstrated, for the first time, the expression of AVT and its receptors in the testes of zebrafish, indicating a direct action of the nonapeptide on testicular function. We demonstrated that AVP decreases the proliferation of pre-meiotic germ cells (type B spermatogonia) while increasing the number of spermatozoa, and that the effect on germ cell development is mediated by androgen. Results support the hypothesis that the short-term action of AVT is to stimulate androgen production, which increases the final stage of spermiogenesis in an androgen-dependent manner. However, prolonged action of AVT can inhibit spermatogenesis by diminishing the proliferation of spermatogonia B, which results in a reduced pool of spermatids and spermatozoa. The present study provides novel information on the physiological significance of vasopressin in vertebrates as a factor involved in the multifactorial control of male reproductive function.

## Figures and Tables

**Table 1:** Primer pairs used for gene expression analysis of testis and brain samples.

Gene	FWD Primer Sequence (5′-3′)	REV Primer Sequence (5′-3′)	GenBank Accession No.	Melting Temp. (°C)	Reference
*eef1a1l1*	AAGACAACCCCAAGGCTCTCA	CCTTTGGAACGGTGTGATTGA	NM_131263.1	59	[26]
*cyp17a*	GGGAGGCCACGGACTGTTA	CCATGTGGAACTGTAGTCAGCAA	NM_212806.3	61	[26]
*fshr*	GAGGATTCCCAGTAATGCTTTCCT	TCTATCTCACGAATCCCGTTCTTC	AY278107.1	60	[45]
*lhr*	CGCTCAGTACCATCCAATGCT	TTGAAGGCATGGCTCTCTATTTCT	AY714133.1	60	[45]
*piwil1*	GATACCGCTGCTGGAAAAAGG	GCAAGACACACTTGGAGAACCA	NM_183338.1	56	[46]
*insl3*	TCGCATCGTGTGGGAGTTT	TGCACAACGAGGTCTCTATCCA	NM_001115053.2	58	[46]
*sycp3*	AGAAGCTGACCCAAGATCATTCC	AGCTTCAGTTGCTGGCGAAA	NM_001040350.1	54.9	[40]
*cimap1b*	GATGCCTGGAGACATGACCAA	CAAAGGAGAAGCTGGGAGCTTT	NM_199958.1	63.4	[29]
*avp*	CGGAGCCCATCAGACAGT	TCGCAGCAGATGCCCTCA	NM_178293.2	56	This paper
*avpr1aa*	CTTCTACGGGCCGGACTTTC	CGGGCTGCTGAGGACTAAACT	NM_001301114.1	58	[4]
*avpr1ab*	CGACTTCTTAGGCTGTTTCC	TAGGCACGCTCTGACTTGAT	NM_001297676.1	58	[4]
*avpr2aa*	CCCGCAGATGTTATGGGATA	AGGCTACCATGATGGGTGTA	XM_001345969.7	57	[4]
*avpr2ab*	TGTGACGAAAGCCATGTCTAAG	GCGGCCCATAACTGAACAATA	XM_001922007.6	57	This paper
*avpr2l*	ATGGGCGCTCAAGCACTAAG	CCGTATGTCAGAGTGGCTTT	NM_001110125.1	58	[4]

## Data Availability

Data will be made available on request.
